# Silencing of ATP Synthase β Impairs Egg Development in the Leafhopper *Scaphoideus titanus*, Vector of the Phytoplasma Associated with Grapevine Flavescence Dorée

**DOI:** 10.3390/ijms23020765

**Published:** 2022-01-11

**Authors:** Matteo Ripamonti, Luca Cerone, Simona Abbà, Marika Rossi, Sara Ottati, Sabrina Palmano, Cristina Marzachì, Luciana Galetto

**Affiliations:** 1 Istituto per la Protezione Sostenibile Delle Piante, Consiglio Nazionale Delle Ricerche, IPSP-CNR, Strada delle Cacce 73, 10135 Torino, Italy; matteo.ripamonti@list.lu (M.R.); cerone_luca@libero.it (L.C.); simona.abba@ipsp.cnr.it (S.A.); marika.rossi@ipsp.cnr.it (M.R.); sara.ottati@ipsp.cnr.it (S.O.); sabrina.palmano@ipsp.cnr.it (S.P.); cristina.marzachi@ipsp.cnr.it (C.M.); 2 Environmental Research and Innovation Department (ERIN), Luxembourg Institute of Science and Technology (LIST), 41 Rue du Brill, 4422 Luxembourg, Luxembourg; 3 Dipartimento di Scienze Agrarie, Forestali ed Alimentari DISAFA, Università degli Studi di Torino, Largo Paolo Braccini 2, Grugliasco, 10095 Torino, Italy

**Keywords:** RNA interference, hexamerin, cathepsin L, RNAi specificity, *Euscelidius variegatus*, female sterility

## Abstract

*Scaphoideus titanus* (Hemiptera: *Cicadellidae*) is the natural vector of Flavescence dorée phytoplasma, a quarantine pest of grapevine with severe impact on European viticulture. RNA interference (RNAi) machinery components are present in *S. titanus* transcriptome and injection of ATP synthase β dsRNAs into adults caused gene silencing, starting three days post injection (dpi) up to 20 dpi, leading to decrease cognate protein. Silencing of this gene in the closely related leafhopper *Euscelidius* *variegatus* previously showed female sterility and lack of mature eggs in ovaries. Here, alteration of developing egg morphology in *S. titanus* ovaries as well as overexpression of hexamerin transcript (amino acid storage protein) and cathepsin L protein (lysosome proteinase) were observed in dsATP-injected females. To evaluate RNAi-specificity, *E.* *variegatus* was used as dsRNA-receiving model-species. Different doses of two sets of dsRNA-constructs targeting distinct portions of ATP synthase β gene of both species induced silencing, lack of egg development, and female sterility in *E. variegatus*, indicating that off-target effects must be evaluated case by case. The effectiveness of RNAi in *S. titanus* provides a powerful tool for functional genomics of this non-model species and paves the way toward RNAi-based strategies to limit vector population, despite several technical and regulatory constraints that still need to be overcome to allow open field application.

## 1. Introduction

*Scaphoideus titanus* Ball (Hemiptera: *Cicadellidae*) is a phloem-feeding monovoltine Nearctic leafhopper, monophagous on grapevine. It was probably introduced accidentally from North America to Europe in mid-19th century, when grapevine materials were brought to France to counteract powdery mildew and *Phylloxera* [1], and population genetics analyses suggest that European populations originated from a single introduction event [2]. *Scaphoideus titanus* was first reported in the late 1950s in France, before spreading throughout the continent, and its current distribution ranges from southern Italy to Hungary and from Portugal to Romania [3]. *Scaphoideus titanus* is the primary vector of Flavescence dorée phytoplasma (FDp), a quarantine pest of grapevine in the EU with a severe impact on European viticulture [4,5]. Phytoplasmas are a group of cell wall-less bacteria with small genome sizes (530–2220 kb) belonging to the class Mollicutes and to date, mainly based on 16Sr genetic data, 47 ‘*Candidatus* Phytoplasma species’ have been described [6,7,8,9]. Phytoplasmas are obligate bacterial plant pathogens, infecting phloem of the host plants as well as the body of insect vectors. These pathogens cause severe symptoms in infected plants, leading to heavy economic losses of many crops worldwide [10,11]. They are transmitted from plant to plant by sap-feeding insect vectors of the order Hemiptera belonging to the suborder Auchenorrhyncha (Fulgoromorpha and Cicadomorpha) and Sternorrhyncha (family Psyllidae) [12]. In the case of FDp, other insect species besides *S. titanus* may transmit the disease, although with a marginal role in secondary spreading of the disease in the vineyard, and are associated with other epidemiological routes [3]. In particular, *Euscelidius variegatus* Kirschbaum (Cicadellidae: Deltocephalinae), a Palearctic, multivoltine and polyphagous species widespread in Europe, North Africa and, introduced in North America, is a laboratory vector of FDp [13] and a natural vector of other phytoplasmas [14], among which is the chrysanthemum yellows strain (CY) of the ‘*Candidatus* Phytoplasma asteris’ [15].

Insecticide control of vector populations is the main strategy to counteract phytoplasma outbreaks [10]. In the case of FD, the currently available control strategies include uprooting of symptomatic grapevines, compulsory insecticide treatments, removal of feral vines, and hot water treatments of rootstocks, scions, or grafted cuttings [4]. More targeted and sustainable pest management approaches are urgently needed to cope with the disease and to overcome the undesirable effects of insecticides on the environment, non-target organisms, and human health. Strategies based on RNA interference (RNAi), originally mainly exploited as a molecular tool for studying insect gene function, are now emerging as a powerful and precise approach to developing new control tactics for insect pests [16,17,18,19]. RNAi is a sequence-specific mechanism naturally occurring in eukaryotes to regulate gene expression and an innate defense against nucleic acids of transposons or viruses [20]. As a molecular tool, RNAi allows specific post-transcriptional silencing of target genes and can be exploited to understand gene function and regulation [21]. RNAi efficiency varies among insect orders, being, for example, very robust in Coleoptera, variable in Diptera, and less efficient in Lepidoptera [16]. Variable efficiencies of this mechanism also occur within species in the same order [19], and many uncertainties remain on RNAi functional requirements in different non-model insects, although the mechanism has been reported for several hemipteran virus vectors [22,23,24,25] as well as for the phytoplasma vector *E. variegatus* [26].

Application of RNAi in crop protection for pest or pathogen control can be achieved by host-induced gene silencing (HIGS), spray-induced gene silencing (SIGS), or virus-induced gene silencing (VIGS) [16]. In any case, high specificity is critical, and off-target effects need to be evaluated and minimized. Indeed, uncertainties concerning dsRNA on non-target species might represent a serious ecological risk for developing dsRNA pesticides for widespread use [27]. For example, high dsRNA sequence identity is required for efficient RNAi in the aphids *Acyrthosiphon pisum* and *Myzus persicae* as well as in the whitefly *Bemisia tabaci* and the mealybug *Planococcus maritimus*, indicating that heterologous dsRNAs at appropriate concentrations may not be a major risk to non-target sap-feeding hemipterans [28]. According to EFSA risk assessment for genetically modified RNAi plants, bioinformatic analyses could help to identify non-target organisms that might be unintentionally affected. Still, laboratory bioassays are needed to assess RNAi effects on non-target species on a case-by-case basis [29].

The present work describes the RNAi machinery of *S. titanus*, sets up optimal parameters to deliver dsRNAs to this species by injection, determines its efficiency at decreasing both transcript and protein levels and evaluates its specificity using different constructs and *E. variegatus* as closely phylogenetic related non-target species. ATP synthase β was selected as the target gene as its silencing in *E. variegatus* decreases survival rate [26], impairs phytoplasma multiplication [30], and induces female sterility by inhibiting oocyte development [31]. Here, we investigated whether a similar effect on egg development also occurs in *S. titanus* females. Interestingly, previous studies demonstrated that ATP synthase β subunit of *E. variegatus* is expressed in mitochondria as well as on the plasma membrane of midgut epithelium and salivary gland cells and interacts in vitro with the antigenic membrane protein of CY phytoplasma [32], which is required for pathogen transmission in vivo [33].

## 2. Results

### 2.1. RNAi Machinery of Scaphoideus titanus

As a first step to understand whether a functional RNAi machinery exists in *S. titanus*, the transcriptome assembly [34] was searched for ten genes involved in the three main insect RNAi pathways [35]: Argonaute1, Dicer1, Loquacious, Drosha, and Pasha (miRNA pathway); Argonaute2, Dicer2, and R2D2 (siRNA pathway); and Argonaute3 and Piwi (piRNA pathway) (Table 1). *Euscelidius variegatus* genes were chosen as queries as this species is phylogenetically close to *S. titanus*, sharing the same feeding habits, which are known to influence the RNAi-response [36]. The presence of all major RNAi pathway genes suggested that a potentially functional RNAi pathway is present in *S. titanus*. The percentage of identity and the query coverage between *S. titanus* proteins and corresponding homologs of *E. variegatus* are listed in Table 1.

### 2.2. ATP Synthase β Is Silenced at Transcript and Protein Levels in Scaphoideus titanus

**Figure 1:**
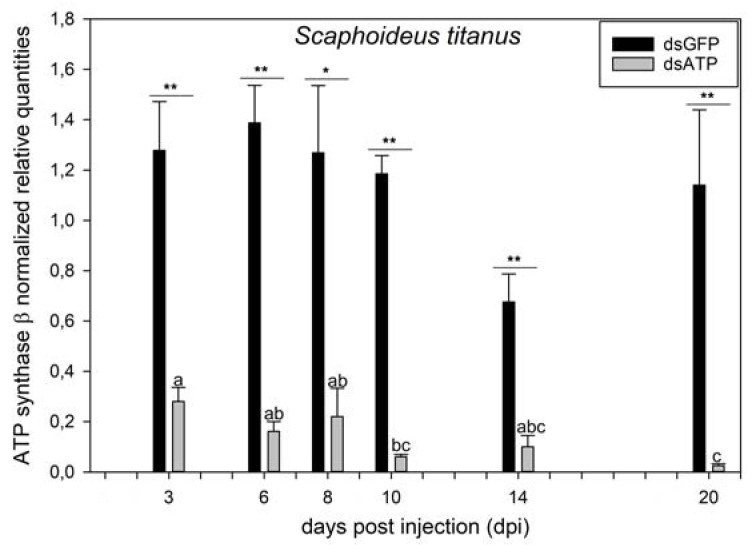
Transcript level of ATP synthase β in *Scaphoideus titanus* injected with dsRNAs. Mean transcript level of ATP synthase β in insects injected with dsRNAs targeting green fluorescent protein (dsGFP) or ATP synthase β (dsATP) at 3, 6, 8, 10, 14, and 20 days post injection (dpi). Within the same date, asterisks indicate significant differences (*t* test) in mean transcript levels of four to five samples ± standard error of the mean (SEM, error bars); * and ** indicate *p* < 0.01 and *p* < 0.001, respectively. Within the same treatment (dsGFP or dsATP), different letters indicate significant differences (ANOVA, followed by the Holm–Sidak method).

The transcript level of ATP synthase β was significantly downregulated at each sampling date from three up to 20 dpi in *S. titanus* specimens injected with 80 ng of St_dsATP1 in comparison with the insects injected with dsGFP (Figure 1). In particular, t values were 4.942, 7.469, 3.284, 15.396, 5.230, and 9.369 at 3, 6, 8, 10, 14, and 20 dpi, respectively, with *p* < 0.001, except at 8 dpi with *p* = 0.007 (Figure 1). Overall abdominal microinjection of ATP synthase β dsRNAs into *S. titanus* adults caused a decrease in the expression of the target gene, with an average reduction ranging from five (3 dpi) to 46 (20 dpi) times in comparison with the corresponding dsGFP-treated controls (Figure 1 and Supplementary Table S1). Transcript levels of ATP synthase β were similar among different sampling dates in insects injected with dsGFP, whereas it significantly decreased at 20 dpi in insects injected with dsATP in comparison with the initial sampling dates (3, 6, and 8 dpi) (one way ANOVA, F = 7.466, *p* < 0.001) (Figure 1).

**Figure 2:**
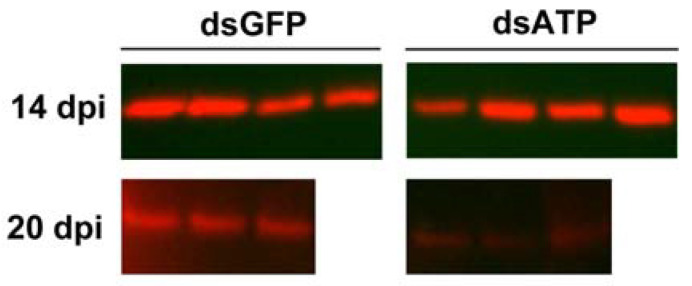
Protein level of ATP synthase β in *Scaphoideus titanus* injected with dsRNAs. Protein expression of ATP synthase β analyzed by western blots on insects (one insect sample per lane) injected with dsRNAs targeting green fluorescent protein (dsGFP) or ATP synthase β (dsATP) at 14 and 20 days post injection (dpi).

Previous results obtained in *E. variegatus* indicated that reduction in ATP synthase β protein level in silenced insects occurred after 15 dpi [30]. Consequently, *S. titanus* injected with dsGFP and dsATP were collected at 14 and 20 dpi and analyzed by western blot to quantify the amount of the corresponding ATP synthase β protein (Figure 2). The anti-ATP synthase β antibody recognized a protein of about 55 kDa (Supplementary Figure S1), as expected from in silico translation of the coding sequence (theoretical pI/*M*_W_: 5.25/55.7 kDa). The protein expression level was similar in the two insect groups at 14 dpi, whereas at 20 dpi, less protein was detected in the extracts from dsATP-injected insects compared with dsGFP-injected ones (Figure 2). Coomassie staining of the same protein samples separated by SDS-PAGE confirmed that equal amounts of proteins from the two insect groups were loaded into gels (Supplementary Figure S1).

### 2.3. Effects of ATP Synthase β Silencing on Survival Rate and Egg Maturation in Scaphoideus titanus

Survival rate of *S. titanus* adults injected either with dsGFP or dsATP was calculated up to 14 dpi in 2019 experiments (Supplementary Figure S2). Two groups of 50 insects were injected with 80 ng of dsGFP (control) and dsATP (construct St_dsATP1), and then monitored after 1, 2, 3, 6, 7, 8, 9, 10, 13, and 14 days. Insects from the two treatments shared similar survival rates (log-rank test, *p* > 0.05).

**Figure 3:**
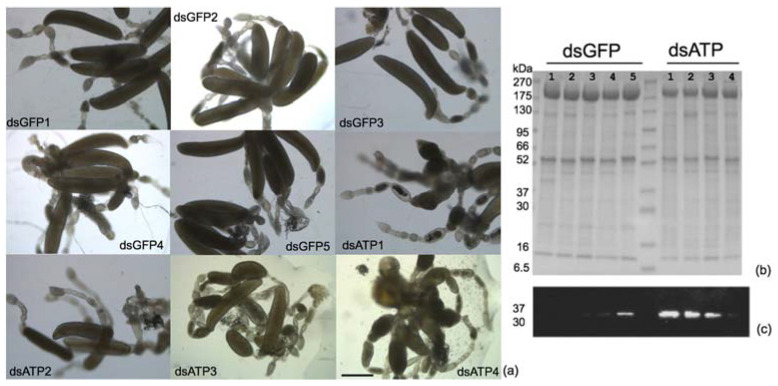
Images of ovaries (**a**) of *Scaphoideus titanus* females collected at 20 days post injection with dsRNAs (80 ng/ insect) targeting green fluorescent protein, (dsGFP1 to 5), used as the control, or ATP synthase β (dsATP1 to 4) observed in bright field. Bar = 500 μm. Mono-dimensional SDS-PAGE (**b**) and corresponding western blots developed with anti-cathepsin L primary antibody (**c**) of total proteins from the same ovary samples observed in (**a**). Molecular weights of Sharpmass VII Prestained Protein Marker (EuroClone) are indicated in KDalton.

Previous results showed that in *E. variegatus*, silencing of ATP synthase β induces female sterility by inhibiting oocyte development concurrently with an overexpression of hexamerin and cathepsin L together with a reduction in vitellogenin in silenced females [31]. Here, the same effects were analyzed in *S. titanus*. Altered oocyte phenotype was observed at 20 dpi in ovaries from silenced females in comparison with dsGFP control samples (Figure 3a). After microscopic observation, the same dissected ovaries analyzed in SDS-PAGE showed an over-expressed band at about 200 kDa in dsGFP samples (Figure 3b) and an over-expression of cathepsin L in three out of four dsATP ovary samples analyzed in western blots (Figure 3c and Supplementary Figure S3). Coomassie staining of the same samples (Figure 3b) confirmed that equal amounts of proteins were loaded into gels.

**Figure 4:**
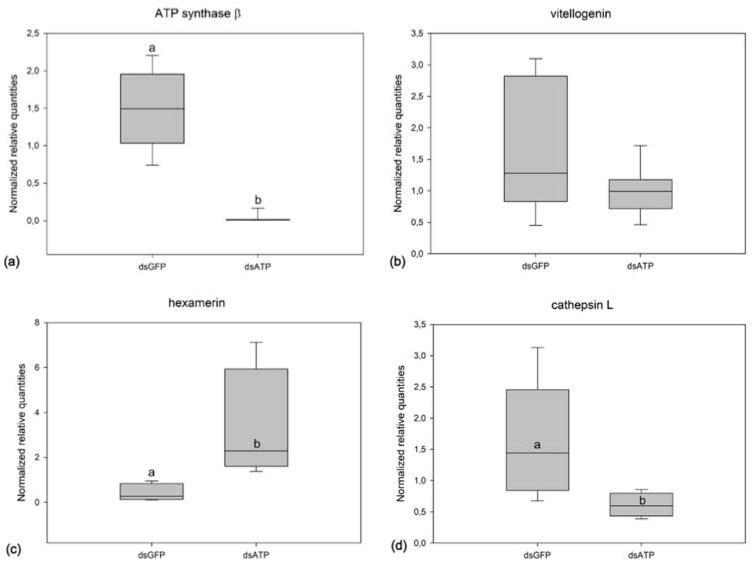
Transcript level of ATP synthase β (**a**), vitellogenin (**b**), hexamerin (**c**), and cathepsin L (**d**) in *Scaphoideus titanus* females (remaining parts of body after dissection of ovaries observed in Figure 3) sampled at 20 days post injection of dsRNAs targeting green fluorescent protein (GFP) or ATP synthase β (St_dsATP1). Different letters indicate significant differences in transcript levels. The median of four to nine samples is shown as a line across each box, the box indicates the 25th and 75th percentiles and whiskers represent the 90th and 10th percentiles.

The expression analysis in remaining parts of the body after the dissection of ovaries from dsGFP- and dsATP-injected females at 20 dpi (Figure 4 and Supplementary Table S2) confirmed a strong silencing of the target ATP synthase β gene, as expected, together with significant upregulation of hexamerin and downregulation of cathepsin L, in silenced females (ATP synthase β: Mann–Whitney T = 50.000, *p* = 0.003; hexamerin: *t* test, *t* = 3.746, *p* = 0.004; cathepsin L: *t* test, *t* = 2.679, *p* = 0.016). The expression of the vitellogenin transcript was not significantly altered in the samples of silenced females.

### 2.4. ATP Synthase β Sequence Analysis

**Figure 5:**
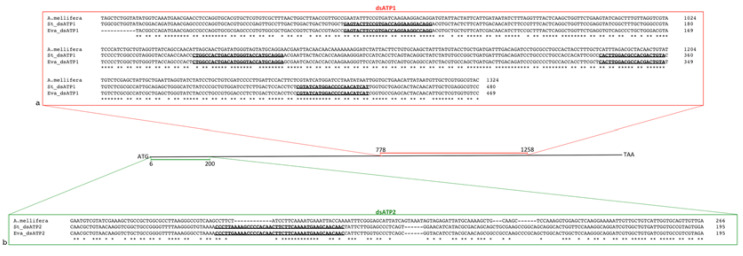
Localization of dsATP1 and dsATP2 on ATP synthase β coding sequence of *Scaphoideus titanus*. Alignments of dsATP1 (**a**) and dsATP2 (**b**) targeting ATP synthase β of *S. titanus* (St_dsATP1 and St_dsATP2) and *Euscelidius variegatus* (Eva_dsATP1 and Eva_dsATP2). ATP synthase β sequence of *Apis mellifera* (XM_006564829.3, LOC551766) was used as the outgroup. Stretch of 21 or more identical nucleotides between *S. titanus* and *E. variegatus* are in bold and underlined.

To evaluate the specificity of silencing induced by treatment with dsRNAs on non-target species, the leafhopper *E. variegatus*, closely related to *S. titanus*, was used as the model dsRNA receiving species. A preliminary analysis was conducted on the coding sequences of ATP synthase β, a very conserved gene through evolution. In particular, the dsATP1 portion, target of the original dsRNA design for *S. titanus* and *E. variegatus* genes, St_dsATP1 (this work) and Eva_dsATP1 [26], was highly conserved in the two leafhoppers as well as in many other insect species, even belonging to distantly related orders, with identities ranging from 79.38 to 86.88% (Table 2). A less conserved portion (dsATP2) of the same gene, with identities ranging from 44.62 to 66.67% (Table 2), was then selected to design new shorter constructs (St_dsATP2 and Eva_dsATP2; Figure 5). Four stretches of 21 or more identical nucleotides between *S. titanus* and *E. variegatus* were present in the dsATP1 portion, whereas only one was found in dsATP2 (Figure 5).

### 2.5. Specificity of dsRNAs on Non-Target Species

**Figure 6:**
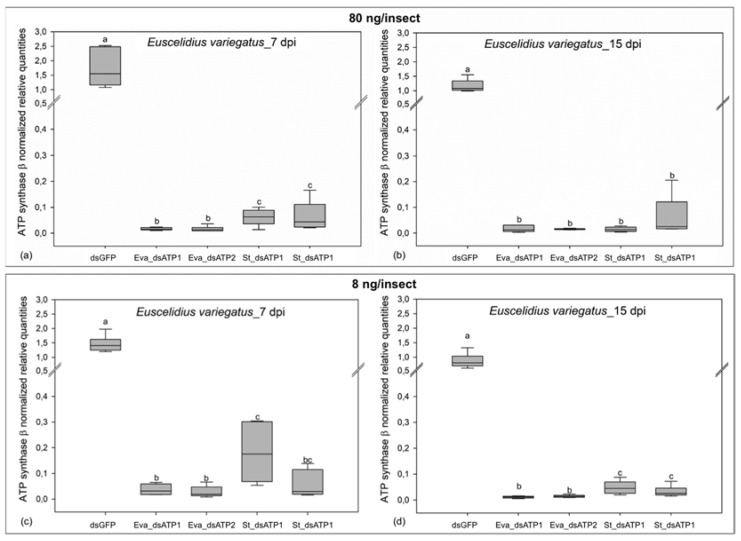
Transcript level of ATP synthase β in *Euscelidius variegatus* injected with different doses and different dsRNA constructs. Transcript level of ATP synthase β in insects injected with 80 (**a**,**b**) and 8 (**c**,**d**) ng/insect of dsRNAs targeting green fluorescent protein (dsGFP) or ATP synthase β of *E. variegatus* (Eva_dsATP1, Eva_dsATP2) and of *Scaphoideus titanus* (St_dsATP1 and St_dsATP2), collected and analyzed at seven (**a**,**c**) and 15 (**b**,**d**) days post injection (dpi). Within the same date and dose, different letters indicate significant differences in transcript levels (ANOVA, followed by Holm–Sidak method). The median of five to seven samples is shown as a line across each box, the box indicates the 25th and 75th percentiles and whiskers represent the 90th and 10th percentiles.

The mean transcript level of ATP synthase β in adults injected with dsRNAs targeting the *E. variegatus* gene was always significantly lower than that measured in insects injected with dsGFP, regardless of the dose (80 or 8 ng/insect), sampling date (7 or 15 dpi), and gene portion used for the dsRNA design (Eva_dsATP1 or Eva_dsATP2) (Figure 6 and Supplementary Table S3). Injection of dsRNAs targeting *S. titanus* ATP synthase β in *E. variegatus* adults induced a significant reduction in the cognate transcript in comparison with dsGFP control insects, regardless of the dose, sampling date, and gene portion (St_dsATP1 or St_dsATP2), even though the silencing effects induced by *S. titanus* dsRNAs were less intense than observed after treatment with *E. variegatus* dsRNAs, especially at the lowest dose of 8 ng/insect (Figure 6 and Supplementary Table S3) (one way ANOVA_80 ng_7 dpi_ F = 63.578, *p* < 0.001; one way ANOVA_80 ng_15 dpi_ F = 35.434, *p* < 0.001; one way ANOVA_8 ng_7 dpi_ F = 34.498, *p* < 0.001; one way ANOVA_8 ng_15 dpi_ F = 99.659, *p* < 0.001).

**Figure 7:**
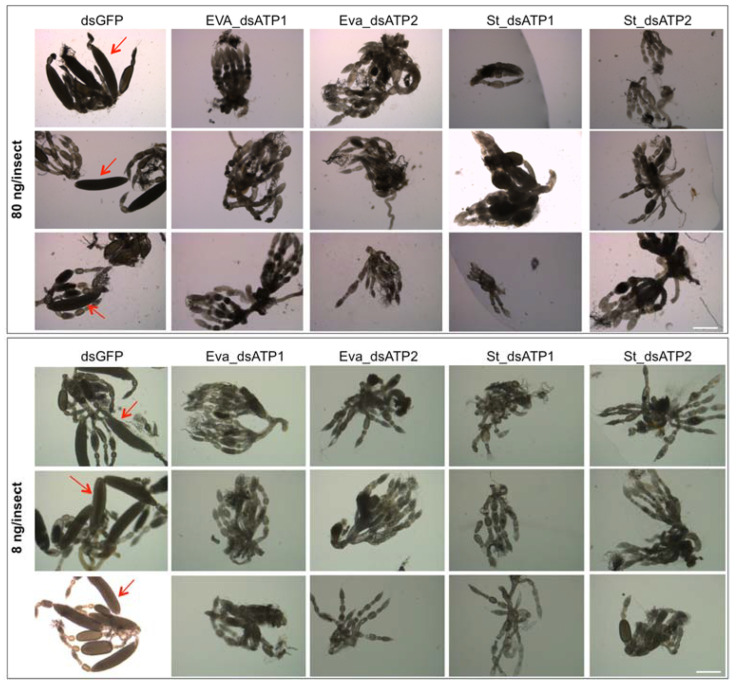
Effects on *Euscelidius variegatus* ovaries following silencing of ATP synthase β. Images of the ovaries of *E. variegatus* adults collected at 15 days post injection with 80 (upper panels) and 8 (lower panels) ng/insect of dsRNAs targeting green fluorescent protein (dsGFP), used as the control, or ATP synthase β of *E. variegatus* (Eva_dsATP1, Eva_dsATP2) and of *Scaphoideus titanus* (St_dsATP1 and St_dsATP2) observed in bright field. Red arrows indicate mature eggs in dsGFP panels. For each treatment and dose, organs from three individuals were observed. Bars = 500 μm.

Silencing of ATP synthase β with the newly designed dsRNAs (Eva_dsATP2, St_dsATP1 and St_dsATP2) caused the alteration of ovaries and the absence/reduction in collected offspring from injected parental *E. variegatus* adults (Figure 7 and Table 3).

## 3. Discussion

In this work, silencing of the ATP synthase β gene of the Hemiptera *Scaphoideus titanus* was achieved through abdominal microinjection of specific dsRNA molecules and the temporal scales of both target transcripts and the cognate protein were verified. Anomalies in egg development were observed in ovaries of *S. titanus* silenced females, together with a significant altered expression of hexamerin and cathepsin L, consistently with previous results obtained in the closely related *E. variegatus* species. The possible cross reactivity of the two leafhoppers to dsRNA triggers tailored on their specific gene sequences was also evaluated.

Since its discovery, RNAi has emerged as an efficient molecular technology for studying gene function. Its application to crop protection is growing rapidly to cope with fungal and viral diseases as well as to limit insect pests [21]. Great expectations are placed in this promising technology due to the easiness of designing powerful tailored solutions and to its intrinsic specificity. Besides genetically modified RNAi plants, the topical use of dsRNAs as biopesticides is a valid alternative to chemical control based on synthetic insecticides such as organophosphates, neonicotinoids, and insect growth regulators [20]. This innovative approach has special implications for invasive insect species that cause severe crop damage despite the extensive use of insecticides, or when alternative non-chemical solutions are often unavailable. For example, in the case of the leafhopper *S. titanus*, vector of the grapevine Flavescence dorée phytoplasma, compulsory insecticide treatments cause unavoidable side effects on the environment and human health and alternative strategies are very limited [37,38,39]. RNAi efficacy in some insects is variable and current knowledge about this mechanism is limited, especially for non-model species. Transcripts of the genes encoding the major components of RNAi machinery were identified here in the transcriptome of *S. titanus* [34]. The gene sets were highly conserved in comparison with the closely related *E. variegatus* species. Among the 10 genes considered, AGO1 proteins of the two leafhoppers showed lower variability than the less conserved AGO2. Indeed, a phylogenetic study on insect AGO proteins indicates a monophyletic origin of AGO1, and confirms that it is usually less variable than AGO2 [40].

Duration of gene silencing induced by exogenous dsRNAs is highly variable among insects. Species of Coleoptera order show strong RNAi responses, which are inherited by the progeny [41], whereas in Diptera and Lepidoptera gene silencing is limited in time and to few cell types [42,43]. Huge variability in the duration of gene silencing has been described within the Hemiptera order ranging from 5–7 days after dsRNA injection for the pea aphid *Acyrthosiphon pisum* [44] to 16 days for the green peach aphid *Myzus persicae* fed on dsRNA-producing transgenic plants [45]. However, the application of RNAi through the injection of dsRNA into few parental adults (parental RNAi) has allowed for the phenotyping of many offspring nymphs in the green rice leafhopper *Nephotettix cincticeps* [25] as well as in the triatomine bug *Rhodnius prolixus* [46], suggesting, at least in these species, a long-lasting silencing effect. In the case of *S. titanus*, silencing of the target gene was measurable at three days after the injection and increased up to 20 days, when the target transcript was reduced over 40 times in comparison with the dsGFP-treated control insects. This is in line with the timing of gene silencing described for *E. variegatus* following dsRNA injection. Indeed, in this species, target transcripts significantly decrease over time [26], silencing is very robust and long-lasting, and it is effective in districts of the insect body far from the injection site [30]. Moreover, the expression level of the two ATP synthase β proteins followed a similar trend in both leafhopper species, showing a sharp decrease after 15 and 20 days post injection of the dsRNAs in *E. variegatus* [30] and *S. titanus* (this work), respectively. 

Proteins belonging to respiratory complexes such as ATP synthase β usually show a slow turnover due to the formation of the mitochondrial respiratory complexes, which require over-transcription of the protein subunits to provide an adequate molecule supply for the assembly process [47]. This may explain the observed delay in the change/alteration of the expression levels of the cognate protein of *S. titanus*. Consistently, no effect on survival rate was observed up to two weeks post injection of the dsATP molecules in *S. titanus*.

As previously observed in *E. variegatus* [31], upon silencing of the ATP synthase β gene, the reproduction of the phytoplasma vector *S. titanus* might be impaired due to the inability to develop mature eggs in ovaries. Silenced females also showed high levels of hexamerin together with low levels of ATP synthase β gene product. Members of the hexamerin family are synthesized in the fat body and then secreted into the hemolymph, where they may accumulate or re-taken up in the fat body and sequestered in granules. Hexamerins are storage proteins, being a source of amino acids and supplying resources for metabolic processes [48]. The levels of these hemolymph storage proteins vary within life stages [49,50], and interestingly, negative correlation between levels of hexamerins and vitellogenin has been observed in the cecropia moth *Hyalophora cecropia*, in the monarch butterfly *Danaus plexippus* [51], and the autogenous mosquito *Aedes atropalpus* [52]. The regular development of eggs and the abundance of a 200 kDa protein (presumably vitellogenin, although in the absence of specific protein identification) observed in ovaries of the dsGFP-injected *S. titanus* females compared to the dsATP samples suggest a negative correlation between the two proteins. The expression of vitellogenin mRNAs in in the remaining parts of the body after ovary dissection did not vary among dsGFP- and dsATP-injected *S. titanus*, suggesting that an altered regulation of vitellogenin may occur at post-transcriptional level or perhaps in the ovaries.

Silenced *S. titanus* females showed a high level of cathepsin L protein in ovaries and a low level of corresponding transcript in the remaining parts of the body after ovary dissection. Previous results from *E. variegatus* showed an over-expression of this gene in the ovaries of silenced females both at the transcript and protein levels [31]. Cathepsins are lysosomal proteases involved in several processes such as development, apoptosis, and immunity of arthropods [53,54]. Interestingly, cathepsin L is specifically involved in oocyte development of the cockroach *Blattella germanica* [55] as well as of the oriental river prawn *Macrobrachium nipponense* [56]. Cathepsin L is therefore considered the most abundant cysteine protease involved in vitellogenesis [56]. These data support the hypothesis that the high level of cathepsin L measured in the ovaries of silenced *S. titanus* females is related to the impairment of egg development.

The abnormal oocyte development, observed in *E. variegatus* following of ATP synthase β silencing with *S. titanus*-specific dsRNAs, confirmed previous results obtained with specific dsRNA triggers, which also induce the mortality of *E. variegatus*, starting from about 10 days after the injection [26], the reduction in phytoplasma multiplication rate [30], and female sterility due to the lack of oocyte development [31]. Although experimental settings to prove this complex phenotype are hard to achieve for the monovoltine and ampelophagous *S. titanus*, this work paves the way to prove the receptor role of selected insect genes intercating in vitro with Flavescence dorée phytoplasma membrane proteins [57].

Specificity of the dsRNA triggers is an important issue when considering the potential application of these molecules in agriculture. ATP synthase β is part of the F1-F0 ATP synthase complex, whose general structure is highly conserved throughout evolution [58]. Analysis of the corresponding coding sequences from 13 insect species belonging to eight orders revealed that the N-terminus portion was less conserved than the C-terminus one. Therefore, we selected two dsRNA constructs specific for each domain of the *S. titanus* and *E. variegatus* genes, and we also tackled a possible dose effect. For each species, gene silencing was always observed following application of the dsRNA triggers, irrespective of their target sequence domain. In the case of *E. variegatus*, the expected sterile phenotype was also always observed, regardless of the administered dsRNA, confirming that this species is very susceptible to RNAi. Moreover, heterologous dsRNAs, targeting the *S. titanus* gene, also triggered RNAi in *E. variegatus*, although the response was less intense than that induced by Eva_dsRNAs, especially at the first sampling date and at the lowest dose. Sequences of both dsATP1 and dsATP2 portions are very similar between the two leafhopper species (>87%), with common stretches of 21 or more identical nucleotides in both regions. This parameter is crucial, as a peak of 21 nucleotide small interfering RNA has been detected in *E. variegatus* following the injection of Eva_dsATP1, indicating the generation of dsRNA-derived siRNAs by the RNAi pathway [30]. The common stretches of 21 or more identical nucleotides may explain the efficient gene silencing in *E. variegatus* treated with *S. titanus* dsRNA. These results may be interesting to better define the relationship between minimum shared nucleotide sequence length and silencing activity. Indeed, a shared 21 nucleotide sequence is required for efficacy against the Western Corn Rootworm *Diabrotica virgifera virgifera* [59], while closely related *Drosophila* species can be selectively controlled with dsRNAs targeting regions of genes with no shared 19–21 nucleotide sequences [60]. However, mutational analyses showed that dsRNAs with >80% sequence identity with target genes trigger RNAi efficiently in various insect species (*Chilo suppressalis*, *Helicoverpa armigera*, *Spodoptera litura*, *Tribolium castaneum*, *Drosophila melanogaster*, and *Locusta migratoria*) and that fragments of ≥16 perfectly matched nucleotides or ≥26 almost perfectly matched ones (with one or two mismatches scarcely distributed) are also able to induce RNAi [27]. In this respect, dsATP2 of *S. titanus* could be a promising portion to avoid off-target effects in species more distantly related than *E. variegatus*, since its identity with other analyzed hemipterans is less than 66%, and even lower with insects belonging to other orders. In this gene portion, homolog-coding sequence of *Apis mellifera* shares at maximum one unique stretch of 12 identical nucleotides with the two leafhopper species. On the other hand, dsATP2 might be useful to control both leafhopper species, which in some cases may co-occur in the same ecological niches, even if ecological risk should be evaluated case by case.

Different RNAi phenotypes are known for Hemiptera, and strategies for the delivery of the silencing triggers, life stage of the insect, selection of the target gene, presence of nuclease degrading the dsRNA trigger, presence, and expression of the core RNAi machinery genes have been listed as key factors to be taken into account at a species-specific level [61]. In particular, delivery of insecticidal dsRNAs to phloem-feeder insects is a major challenge for RNAi application in crop protection, although several solutions have been recently proposed [18,62,63,64,65]. In the hypothesis of the topical application of dsRNAs to plants, foliar spray may not be the best choice, as many physical (cuticle, cell wall) and biochemical (nucleases) barriers strongly inhibit successful uptake of these molecules by the phloem feeder [66]. On the other hand, dsRNA application to the plant by root and/or petiole absorption leads to systemic translocation of the trigger through the xylem [67,68,69]. Although *S. titanus* is primarily a phloem feeder, analysis of its electrical penetration graph indicates that the insect feeds on both vascular tissues, spending time feeding on the xylem [70,71], as commonly also observed for other leafhoppers [72,73,74,75].

Although there are high expectations for the use of RNAi to control insect pest populations in a very specific way, we need to explore the effects and constraints this technique may experience when applied to agronomical important pests beside model organisms. This work tackles two non-model hemipteran species, both vectors of phytoplasma diseases to ornamental and fruit crops. Finally, lack of harmonized regulatory legislation among different countries highlights the urgent need to develop appropriate science-based risk assessment procedures for RNAi applications [29,76]. To this purpose, *E. variegatus* can be proposed as a model insect closely related to *S. titanus* for off-target bioassays in the risk assessment plans of RNAi-based strategies.

## 4. Materials and Methods

### 4.1. Insect Rearing

*Scaphoideus titanus* has one generation per year and its continuous rearing under controlled conditions is not feasible. To obtain coeval *S. titanus* specimens, two-year-old grapevine canes bearing leafhopper eggs were collected in Piedmont (Italy) infested vineyards during the winter period and kept at 5 ± 1 °C. To allow a coordinated egg hatching, grapevine branches were caged inside insect-proof screen houses in a glasshouse with natural light and temperature ranging from 20 to 25 °C. Potted grapevine (*Vitis vinifera* L.) cuttings and healthy broad bean (*Vicia faba* L.) plants from seed were introduced in the screen house to feed the newly hatched nymphs. Insect adults were collected and used for dsRNA injection. Adults emerged in 2019 were used for over time evaluation of ATP synthase β transcript and protein levels as well as to calculate survival rate up to 14 days post injection (dpi). Insects emerged in 2020 were used to check transcript and protein levels at 20 dpi, whereas females emerged in 2021 were used to observe the silencing effect on egg development in ovaries.

*Euscelidius variegatus* was used as non-target species to check the specificity of dsRNA treatments. The species was originally collected in Piedmont and continuously reared on oat, *Avena sativa* (L.), inside plastic and nylon cages in growth chambers at 20–25 °C with a L16:D8 photoperiod. To obtain coeval newly emerged *E. variegatus* adults, about two weeks before each experiment, the required amount of 4^th^ and 5^th^ instar nymphs were caged together on oats, separately from the main rearing, and then used for dsRNA injection once they were adults.

### 4.2. Synthesis and Delivery of dsRNA

The complete coding sequence of *S. titanus* ATP synthase β (GenBank accession number: MZ130944) was retrieved from a transcriptome project aimed at describing the virus population of this insect species [34]. The complete coding sequence of *E. variegatus* ATP synthase β (target mRNA) can be found in the TSA sequence database (BioProject: PRJNA393620) at NCBI under the accession number GFTU01013594.1.

Fragments of the target sequences were obtained from total RNA isolated from adult insects using a reverse transcription polymerase chain reaction (RT-PCR). A control template corresponding to a fragment of the gene sequence of green fluorescent protein (GFP), surely absent in insect genomes, was PCR-amplified from plasmid pJL24 [77]. Primers used to generate the dsRNA templates included the T7 promoter sequence at the 5′-end (Supplementary Table S4). The PCR products were then ligated into the pGEM-T Easy plasmid (Promega, Madison, WI, USA) and the plasmids were used as templates for the subsequent PCRs. Then, 1 μg of each column-purified PCR product was in vitro transcribed using the MEGAscript RNAi Kit (Thermo Fisher Scientific, Waltham, MA, USA) according to the manufacturer’s instructions. After column-purification with ssDNA/RNA Clean and Concentrator (Zymo Research, Irvine, CA, USA) and elution in Tris–EDTA buffer (10 mM Tris–HCl, 0.1mM EDTA, pH 8.5), dsRNAs were quantified using a Nanodrop ND-1000 spectrophotometer (Thermo Fisher Scientific).

Newly emerged adults were anesthetized with CO_2_ and microinjected between two abdominal segments under a stereomicroscope using a fine glass needle connected to a Cell Tram Oil microinjector (Eppendorf, Hamburg, Germany). Adults of *S. titanus* were microinjected with 0.5 μL of dsRNAs (dsGFP and St_dsATP1) at the concentration of 160 ng μL^−1^ (80 ng per insect). For specificity assays, adults of *E. variegatus* received decreasing doses (80 and 8 ng per insect) of different dsRNAs. Groups of injected insects were then caged on potted grapevines (*S. titanus*) or on oat plants (*E. variegatus*) and monitored daily until the end of the experiments. Dead insects were removed periodically.

### 4.3. Ovary Observation and Progeny Collection

Previous results demonstrated that silencing of ATP synthase β induces female sterility in *E. variegatus* [31]. To evaluate the phenotypic effects on egg development before deposition, ovaries were collected from injected females of *S. titanus* and *E. variegatus*, caged soon after the injection with untreated males of corresponding species. At 15 (*E. variegatus*) and 20 dpi (*S. titanus*), insects were anesthetized with CO_2_ and organs were dissected with forceps and needles using a stereomicroscope in a drop of phosphate-buffered saline (PBS), mounted on glass slides and observed under a DM750 (Leica Wetzlar, Germany) microscope equipped with a CoolLED pE300 white Illumination System. Images were acquired with a Leica EC4 camera controlled through the LAS EZ software. After dissection and observation of ovaries from *S. titanus* females, the remaining parts of the body as well as observed ovaries were immediately separately frozen in liquid nitrogen for the further extraction of RNA and proteins, respectively.

To evaluate fecundity of *E. variegatus*, groups of dsRNA injected insects were caged on oats for 15 days and, about at 60 dpi, offspring was collected and counted. Mean ratio of offspring per parental female was calculated by dividing the total number of collected F1 specimens by the number of parental females collected alive at the end of the ovoposition period.

### 4.4. RNA Extraction, cDNA Synthesis, and Gene Expression Analysis

Total RNAs were extracted from single *S. titanus* or *E. variegatus* adults following dsRNA injection. The samples were frozen in liquid nitrogen, crushed with a micropestle in sterile Eppendorf tubes, and homogenized in 0.5 mL Tri-Reagent (Zymo Research). Samples were centrifuged 1 min at 12,000× *g* at 4 °C and RNAs were extracted from supernatants with the Direct-zol RNA Mini Prep Kit (Zymo Research), following the manufacturer’s protocol and including the optional DNAse treatment step. Concentration, purity, and quality of extracted RNA samples were analyzed in a Nanodrop ND-1000 spectrophotometer (Thermo Fisher Scientific).

Quantitative RT-PCR (qRT-PCR) was used to quantify the ability of the injected dsRNAs to knockdown target mRNA (ATP synthase β) and to measure the effect on the transcript level of vitellogenin (GenBank accession: OL692454), hexamerin (GenBank accession: OL692455) and cathepsin L (GenBank accession: OL692453) of *S. titanus*. Four to nine biological replicates were analyzed at each time point for each dsRNA-injected group. For each sample, cDNA was synthesized from total RNA (1 μg) with random hexamers using a High Capacity cDNA Reverse Transcription Kit (Thermo Fisher Scientific). The resulting cDNA was used as a template for qPCR in a 10 μL volume mix, containing 1 × iTaq Universal Sybr Green Supermix (Bio-Rad, Hercules, CA, USA) and 300 nM of each primer. All the primer pairs used for qPCR are listed in Supplementary Table S4. Samples were run in duplicate in a CFX Connect Real-Time PCR Detection System (Bio-Rad). Cycling conditions were: 95 °C for 3 min, and 40 cycles at 95 °C for 15 s, and 60 °C for 30 s of the annealing/extension step. The specificity of the PCR products was verified by melting curve analysis for all samples. No-template controls were always included in each plate. Primers targeting glutathione S-transferase (GenBank accession number: MZ130943) and elongation factor-1α (GenBank accession number: MZ130942) were used as reference genes to normalize the cDNA among samples. Normalized expression levels of each target gene for each sample were calculated by CFXMaestro™ Software (Bio-Rad). The expression stability of reference genes was acceptable in the multiplate gene study.

### 4.5. Protein Gels and Western Blots

The following procedure was used to quantify the ability of the injected dsRNAs to reduce the expression of the ATP synthase β protein, encoded by the corresponding silenced target gene, in *S. titanus* specimens collected at 14 and 20 dpi. Single insects were homogenized in a 1.5 mL tube with a micro-pestle in 100 μL of Rx Buffer (0.1% Triton X-100, 100 mM KCl, 3 mM NaCl, 3.5 mM MgCl_2_, 1.25 mM EGTA, and 10 mM Hepes, pH 7.3) [78], sonicated for 1 min at RT, and centrifuged for 1 min at 13,000× *g*. The supernatant was recovered and an aliquot was quantified in a UV–Vis spectrophotometer with Bradford reagent (Bio-Rad) along a standard scale based on known dilutions of bovine serum albumin (BSA, Sigma-Aldrich, St. Louis, MO, USA) dissolved in Rx Buffer. For each sample, 1 μg of total proteins was loaded onto a 4–20% polyacrylamide TGX pre-cast gel (Bio-Rad), together with 3 μL of Sharpmass VII Prestained Protein Marker (EuroClone, Milan, Italy). Gels were either stained with colloidal Coomassie stain [79] or blotted on a polyvinylidene difluoride (PVDF) membrane. Coomassie stain was applied to further confirm that the amount of total proteins was equivalent among the different loaded samples. Membranes were blocked for 30 min with 3% BSA dissolved in Tris-buffered saline with 0.1% Tween (BSA-TBST) and incubated overnight at 4 °C with primary antibody (anti-ATP synthase β AS05 085 rabbit-developed, Agrisera, Vännäs, Sweden) diluted 1:10,000 in BSA-TBST. Blots were then washed four times with BSA-TBST, incubated for 2 h with horseradish peroxidase (HRP)-conjugated secondary antibody (A0545 anti-rabbit goat-developed, Sigma-Aldrich) diluted 1:15,000 in BSA-TBST, washed four times with TBST, and developed with Pierce West Pico SuperSignal chemiluminescent substrate (Thermo Fisher Scientific) in a VersaDoc 4000 MP system (Bio-Rad).

The same procedure with slight modifications was used to evaluate the expression of cathepsin L in ovaries dissected from *S. titanus* silenced females collected at 20 dpi. Briefly, organs from a single insect were separately homogenized in 30 μL of Rx Buffer and sonicated for 1 min. Protein extracts were quantified as above, and 0.4 μg of total proteins was loaded onto gels either stained or blotted. After blocking, blots were incubated with primary antibody (anti-cathepsin L ab200738 rabbit-developed, Abcam, Cambridge, UK) diluted 1:1000 in BSA-TBST, and then washed, incubated with secondary antibody, and developed as detailed above.

### 4.6. Statistical Analyses

SigmaPlot version 13 (Systat Software, Inc., San Jose, CA, USA) was used for statistical analyses. As raw transcript data were not always normally distributed, they were natural log-transformed before analysis. The *t*-test was used to compare transcript levels measured within the same date in *S. titanus* injected with dsGFP or dsATP. ANOVA, followed by the Holm–Sidak method as a multiple comparison procedure, was used to compare transcript levels measured over time within the same treatment in *S. titanus* and within the same date in *E. variegatus* injected with the same dose of different dsRNAs. Kaplan–Meier analysis was used to estimate the survival up to 14 dpi of *S. titanus* injected with dsGFP or St_dsATP1, considering that insects could die (event of interest) or be censored (i.e., sampled at different time points for expression analyses). *T* test or Mann–Whitney test, when the parametric analysis assumptions were not met, were used to compare levels of different transcripts measured in the remaining parts of the body after ovary dissection from the injected *S. titanus* females.

## Figures and Tables

**Table 1:** Names and NCBI accession numbers of the genes involved in the insect RNAi pathways. Percentages of sequence identities and query coverage between *Scaphoideus titanus* RNAi pathway proteins and the corresponding proteins from *Euscelidius variegatus* are indicated.

Gene Names	*Scaphoideus* *titanus*	*Euscelidius* *variegatus* ^1^	Percent Identity	Query Coverage
AGO1	MZ161181	GFTU01004640.1	99	99
AGO2	MZ161183 ^2^	GFTU01008426.1 ^2^	85	92
AGO3	MZ161185	GFTU01016377.1 ^2^	86	84
Dicer1	MZ161180 ^2^	GFTU01004888.1 ^2^	90	99
Dicer2	MZ161186	GFTU01010822.1	70	99
Drosha	MZ161187	GFTU01002931.1 ^2^	67	71
Loquacious	MZ161182	GFTU01010148.1	84	100
Pasha	MZ161189	GFTU01010211.1	80	97
Piwi	MZ161188	GFTU01014046.1	88	100
R2D2	MZ161184	GFTU01005523.1	65	99

^1^ *Euscelidius variegatus* accession numbers are referred to TSA sequence database (BioProject: PRJNA393620). ^2^ Partial sequences.

**Table 2:** Percent identities of sequences encoding ATP synthase β of different insect species aligned with St_dsATP1 and St_dsATP2 targeting ATP synthase β of *Scaphoideus titanus*.

Insect Species	Order	Percent Identity with St_dsATP1	Percent Identity with St_dsATP2
*Euscelidius variegatus*	Hemiptera	87.63	88.72
*Metcalfa pruinosa*		84.79	66.15
*Halyomorpha halys*		83.12	66.67
*Frankliniella occidentalis*	Thysanoptera	86.88	55.90
*Helicoverpa armigera*	Lepidoptera	86.88	51.85
*Ostrinia furnacalis*		84.79	44.62
*Nasonia vitripennis*	Hymenoptera	85.42	55.19
*Apis mellifera*		79.38	58.62
*Locusta migratoria*	Orthoptera	85.00	54.26
*Dendroctonus ponderosae*	Coleoptera	84.58	51.55
*Zootermopsis nevadensis*	Dictyoptera	82.92	55.38
*Drosophila melanogaster*	Diptera	81.25	47.02

*S. titanus:* MZ130944; *E. variegatus*: GFTU01013594.1 (BioProject: PRJNA393620); *M. pruinosa*: GDFH01046050.1 (C229983_a_55_0_l_2332); *H. halys*: XM_014424491.1 (LOC106683194); *F. occidentalis*: XM_026438717.1 (LOC113218387); *H. armigera*: XM_021333195.1 (LOC110375167); *O. furnacalis*: XM_028312547.1 (LOC114358553); *N. vitripennis*: NM_001159894.1; *A. mellifera*: XM_006564829.3 (LOC551766); *L. migratoria*: KX357709.1; *D. ponderosae*: XM_019908089.1 (LOC109539986); *Z. nevadensis*: XM_022079501.1 (LOC110837409); *D. melanogaster*: NM_166808.3.

**Table 3:** Effects of ATP synthase β silencing on *Euscelidius variegatus* progeny. Numbers of offspring of *E. variegatus* following the injection of newly emerged F0 parent adults with different doses of dsRNAs targeting green fluorescent protein (GFP) or different portions of ATP synthase β gene of *E. variegatus* (Eva_dsATP1, Eva_dsATP2) and of *Scaphoideus titanus* (St_dsATP1, St_dsATP2). Groups of injected F0 females and males listed in the same line were caged together for 15 days right after the dsRNA injection. Numbers of males and females collected alive at the end of this period are indicated. Offspring insects (F1, nymphs, and adults) were collected 60 days post injection.

dsRNA Injected	Dose of dsRNA (ng/insect)	N° F0 Parental Insects		N° F1 Offspring		Mean Ratio of Offspring/Parental Female
		Males	Females	Nymphs	Adults	
dsGFP	80	2	12	80	2	6.83
	8	5	18	128	18	8.11
Eva_dsATP1	80	1	6	0	0	-
	8	4	9	0	5	0.55
Eva_dsATP2	80	1	5	0	0	-
	8	4	6	0	0	-
St_dsATP1	80	5	8	0	0	-
	8	4	8	0	4	0.50
St_dsATP2	80	1	8	0	0	-
	8	2	3	2	0	0.67

## Supplementary Materials

The following are available online at https://www.mdpi.com/article/10.3390/ijms23020765/s1.
