# Supplementary material for: Silencing of ATP Synthase β Impairs Egg Development in the Leafhopper Scaphoideus titanus, Vector of the Phytoplasma Associated with Grapevine Flavescence Dorée
Source: Int J Mol Sci. 2022 Jan 11;23(2):765. doi: 10.3390/ijms23020765 (PMC8775575; doi:10.3390/ijms23020765)
Supplement: Supplementary file 1 [file ijms-23-00765-s001.zip › ijms-1526715-supplementary.pdf]

Article

Silencing of ATP synthase β impairs egg development in the leafhopper *Scaphoideus titanus*, vector of the phytoplasma associated with grapevine Flavescence dorée

Matteo Ripamonti[1], Luca Cerone[1,2], Simona Abbà[1], Marika Rossi[1], Sara Ottati[1,2], Sabrina Palmano[1], Cristina Marzachì[1] and Luciana Galetto[1,*]

[1] Istituto per la Protezione Sostenibile Delle Piante, Consiglio Nazionale Delle Ricerche, IPSP-CNR, Strada delle Cacce 73, 10135 Torino, Italy; matteo.ripamonti@list.lu (M.R.); cerone_luca@libero.it (L.C.); simona.abba@ipsp.cnr.it (S.A.); marika.rossi@ipsp.cnr.it (M.R.); sara.ottati@ipsp.cnr.it (S.O.); sabrina.palmano@ipsp.cnr.it (S.P.); cristina.marzachi@ipsp.cnr.it (C.M.)

[2] Environmental Research and Innovation Department (ERIN), Luxembourg Institute of Science and Technology (LIST), 41 Rue du Brill, 4422 Luxembourg, Luxembourg

[3] Dipartimento di Scienze Agrarie, Forestali ed Alimentari DISAFA, Università degli Studi di Torino, Largo Paolo Braccini 2, Grugliasco, 10095 Torino, Italy

* Correspondence: luciana.galetto@ipsp.cnr.it

Supplementary Tables

Supplementary Table S1. Over time expression of ATP synthase β transcript in *Scaphoideus titanus* following dsRNA injection. Mean ± standard error of the mean (SEM), total number of samples (N), maximum/minimum and median of normalized relative expression of ATP synthase β transcript measured in *Scaphoideus titanus* sampled at 3, 6, 8, 10 and 14 days post injection of dsRNAs targeting green fluorescent protein (GFP) or ATP synthase β (St_dsATP1).

Days post injection	Target of dsRNAs	ATP synthase β expression		
		**Mean ± SEM (N)**	**Max/Min**	**Median**
3	GFP	1.278 ± 0.194 (5)	1.928/0.796	1.250
	ATP synthase *β*	0.280 ± 0.056 (5)	0.477/0.125	0.258
6	GFP	1.387 ± 0.149 (5)	1.956/1.113	1.235
	ATP synthase *β*	0.280 ± 0.038 (5)	0.289/0.049	0.145
8	GFP	1.268 ± 0.268 (5)	2.110/0.656	1.028
	ATP synthase *β*	0.220 ± 0.113 (4)	0.557/0.081	0.120
10	GFP	1.185 ± 0.072 (5)	1.327/1.034	1.093
	ATP synthase *β*	0.060 ± 0.009 (5)	0.083/0.035	0.059
14	GFP	0.676 ± 0.111 (4)	0.952/0.408	0.672
	ATP synthase *β*	0.101 ± 0.045 (5)	0.272/0.022	0.080
20	GFP	1.140 ± 0.299 (4)	1.894/0.573	1.047
	ATP synthase *β*	0.025 ± 0.007 (4)	0.045/0.011	0.023

Supplementary Table S2. Expression of selected transcripts related with egg development in *Scaphoideus titanus* females following dsRNA injection. Mean ± standard error of the mean (SEM), total number of samples (N), maximum/minimum and median of normalized relative expression of ATP synthase β, vitellogenin, hexamerin and cathepsin L transcripts measured in *Scaphoideus titanus* females (remaining parts of body after dissection of ovaries observed in Figure 3) sampled at 20 days post injection of dsRNAs targeting green fluorescent protein (GFP) or ATP synthase β (St_dsATP1).

**Target genes**	**Target of dsRNAs**	**Normalized relative quantities**		
		**Mean ± SEM (N)**	**Max/Min**	**Median**
ATP synthase *β*	GFP	1.493 ± 0.239 (5)	2.203/0.738	1.491
	ATP synthase *β*	0.033 ± 0.022 (7)	0.168/0.005	0.013
vitellogenin	GFP	1.717 ± 0.482 (5)	3.098/0.450	1.280
	ATP synthase *β*	0.977 ± 0.124 (9)	1.721/0.459	0.991
hexamerin	GFP	0.441 ± 0.169 (5)	0.956/0.118	0.278
	ATP synthase *β*	3.273 ± 1.302 (4)	7.125/1.383	2.291
cathepsin L	GFP	1.608 ± 0.424 (5)	3.131/0.673	1.440
	ATP synthase *β*	0.609 ± 0.096 (4)	0.857/0.390	0.594

Supplementary Table S3. Over time expression of ATP synthase β transcript in *Euscelidius variegatus* following injection of different constructs of dsRNAs. Mean ± standard error of the mean (SEM), total number of samples (N), maximum/minimum and median of normalized relative expression of ATP synthase β transcript measured in *Euscelidius variegatus* sampled at 7 and 15 days post injection of 80 and 8 ng per insect of dsRNAs targeting green fluorescent protein (GFP) or ATP synthase β of *E. variegatus* (Eva_dsATP1, Eva_dsATP2) and of *Scaphoideus titanus* (St_dsATP1 and St_dsATP2).

**Dose of dsRNAs (ng/insect)**	**Days post injection**	**Target of dsRNAs**	**ATP synthase β expression**		
			**Mean ± SEM (N)**	**Max/Min**	**Median**
80	7	GFP	1.727 ± 0.256 (6)	2.522/1.076	1.553
		Eva_dsATP1	0.017 ± 0.002 (6)	0.024/0.010	0.015
		Eva_dsATP2	0.016 ± 0.004 (6)	0.036/0.009	0.013
		St_dsATP1	0.061 ± 0.013 (6)	0.100/0.014	0.063
		St_dsATP2	0.065 ± 0.023 (6)	0.166/0.020	0.044
	15	GFP	1.159 ± 0.102 (5)	1.557/0.989	1.076
		Eva_dsATP1	0.017 ± 0.006 (5)	0.031/0.003	0.012
		Eva_dsATP2	0.015 ± 0.001 (5)	0.019/0.013	0.015
		St_dsATP1	0.014 ± 0.004 (5)	0.027/0.004	0.014
		St_dsATP2	0.060 ± 0.037 (5)	0.206/0.016	0.025
8	7	GFP	1.459 ± 0.113 (6)	1.977/1.199	1.406
		Eva_dsATP1	0.037 ± 0.009 (5)	0.065/0.017	0.031
		Eva_dsATP2	0.023 ± 0.009 (6)	0.066/0.008	0.019
		St_dsATP1	0.203 ± 0.068 (6)	0.512/0.054	0.175
		St_dsATP2	0.060 ± 0.024 (5)	0.138/0.016	0.029
	15	GFP	0.871 ± 0.102 (6)	1.326/0.624	0.805
		Eva_dsATP1	0.011 ± 0.002 (5)	0.016/0.005	0.011
		Eva_dsATP2	0.015 ± 0.002 (7)	0.023/0.009	0.014
		St_dsATP1	0.048 ± 0.010 (6)	0.088/0.020	0.045
		St_dsATP2	0.033 ± 0.008 (6)	0.072/0.015	0.026

Supplementary Table S4. List of primers used in this work.

Application	Primer name	5′-3′ sequences	Organism/ target gene	Prod. size	qPCR efficiency	R^2	Melt peak
dsRNA synthesis	St_T7ATPsynBetaf[#]	TAATACGACTCACTATAGTGGCGCTGGTATACGGACAG	*S. titanus*/ ATP synthase *β*	480 nt	/	/	/
	St_T7ATPsynBetar[#]	TAATACGACTCACTATAGGGACGCCTCGAGCAATGTTG					
	St_T7ATPsynBetaf_2[#]	TAATACGACTCACTATAGCAACGCTGTAACAAGGTCGG		195 nt	/	/	/
	St_T7ATPsynBetar_2[#]	TAATACGACTCACTATAGTCCACTACGGCACCAATCAC					
	Eva_T7ATPsynBetaf[1]	TAATACGACTCACTATAGTACGGCCAGATGAACGAGCC	*E. variegatus*/ ATP synthase *β*	469 nt	/	/	/
	Eva_T7ATPsynBetar[1]	TAATACGACTCACTATAGGGACACCACGAGCAATGTTG					
	Eva_T7ATPsynBetaf_2[#]	TAATACGACTCACTATAGCAACGCTGTAACAAGGTCTG		195 nt	/	/	/
	Eva_T7ATPsynBetar_2[#]	TAATACGACTCACTATAGTCTACGACGGCACCGATCAC					
	T7GFPf[1]	TAATACGACTCACTATAGCTTTTCACTGGAGTTGTCCC	GFP	403 nt	/	/	/
	T7GFPr[1]	TAATACGACTCACTATAGGTTTGTGTCCGAGAATGTTTC					
qPCR target genes	St_ATPβFw622[#]	CGTTTCACTCAGGCTGGTTC	*S.titanus*/ ATP synthase *β*	171 nt	85.4%	0.999	82.5°C
	St_ATPβRv792[#]	ATCATCAGCCGGCACATAGA					
	St_Vitel_F[#]	AAGAGGAACATGCGCTCCTA	*S.titanus*/ vitellogenin	98 nt	96.2%	0.998	78.0°C
	St_Vitel_R[#]	TTGCTGGGCAGGAAACTATC					
	St_HEX_F710[#]	ACGCCAACCAGTACCAGAAC	*S.titanus*/ hexamerin	115 nt	99.4%	0.996	78.5°C
	St_HEX_R824[#]	GCAGGGTTCTTCATCTGCTC					
	St_CathL473_F[#]	CATCAAGGACCAGGGCCACTG	*S.titanus*/ cathepsin L	92 nt	95.7%	0.998	82.0°C
	St_CathL473_R[#]	CCAGCTTTCCTGACTTGCGG					
	ATPβFw622[2]	CGCTTTACTCAGGCTGGTTC	*E.variegatus*/ ATP synthase *β*	171 nt	100%	0.995	84.5°C
	ATPβRv792[2]	GTCATCAGCTGGCACGTAGA					
qPCR reference genes	St_EF1-*α*_F215[#]	CCATTGACATTGCCCTGTGG	*S.titanus*/ elong. factor-1*α*	110 nt	98.0%	0.999	77.5°C
	St_EF1-*α*_R325[#]	CCTGAGAAGTTCCAGTAATCATG					
	St_GST1_F257[#]	CTAAGGATGCCCAGAAACGA	*S.titanus*/ glut. S-transfer.	113 nt	94.5%	0.998	79.0°C
	GST1_R369[3]	TGGCGCTCCTCCAAACATCA					
	EF1-*α*_F215[3]	CCATCGACATTGCCCTGTGG	*E.variegatus*/ elong. factor-1*α*	111 nt	87.9%	0.997	78.5°C
	EF1-*α*_R325[3]	CCTGTGAGGTTCCAGTGATCATG					
	GST1_F257[3]	CCAAGGACCCCAAGAAGCGA	*E.variegatus*/ glut. S-transfer.	113 nt	87.3%	0.997	79.0°C
	GST1_R369[3]	TGGCGCTCCTCCAAACATCA					

T7 promoter sequence is underlined.

[#]Primers designed in this work.
[1] Abbà et al., 2019
[2] Galetto et al., 2013
[3] Galetto et al., 2018

Supplementary Figures

Supplementary Figure S1

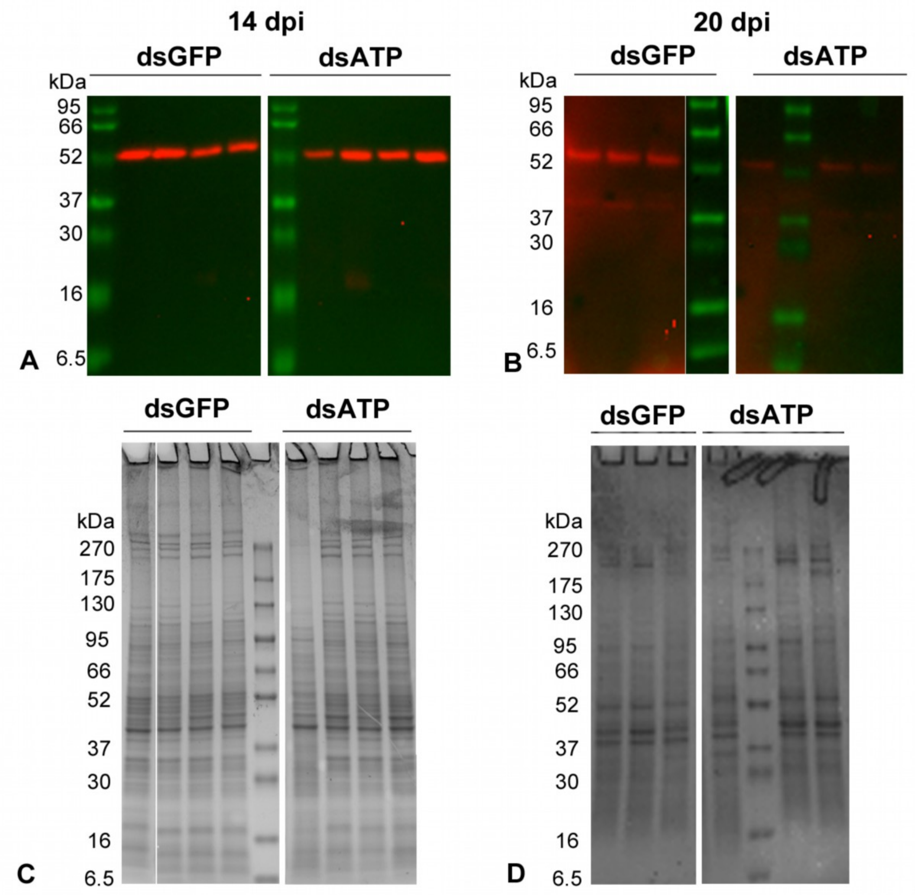

Supplementary Figure S1. **Full lenght images of Western blots depicted in Figure 2 and corresponding SDS-PAGE.** Full lenght images of Western blots (WB) (upper panels, A and B) depicted in Figure 2, developed with anti-ATP synthase β antibody, and corresponding Coomassie stained SDS-polyacrylamide gels (lower panels, C and D), run in parallel with WB on the same *Scaphoides titanus* insect samples (one insect sample per single lane), analyzed at 14 (panels A and C) and 20 (panels B and D) days post injection (dpi) of dsRNAs targeting green fluorescent protein (dsGFP) or ATP synthase β (dsATP), with construct St_dsATP1. The masses of protein standards migrated together with samples are indicated in kDa.

Supplementary Figure S2

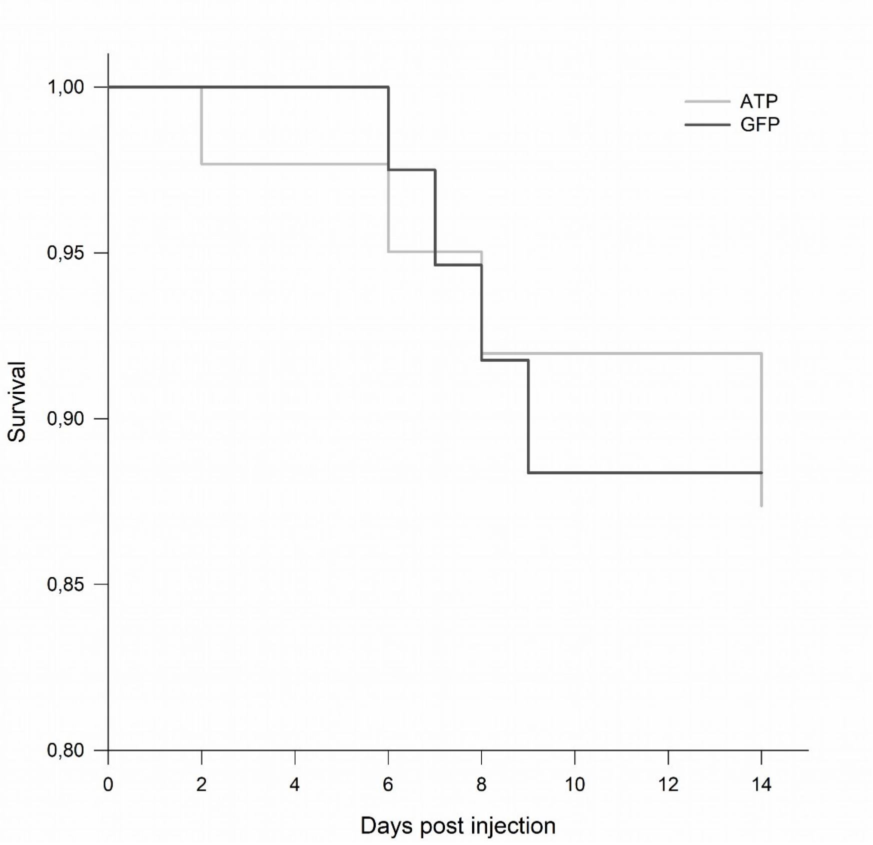

Supplementary Figure S2. **Survival rates of *Scaphoideus titanus* following dsRNA injection.** Survival rates of 61 *Scaphoideus titanus* adults over a 14-day period after microinjection of 80 ng of dsRNAs targeting green fluorescent 62 protein (GFP) or ATP synthase β (ATP), with construct St_dsATP1.

Supplementary Figure S3

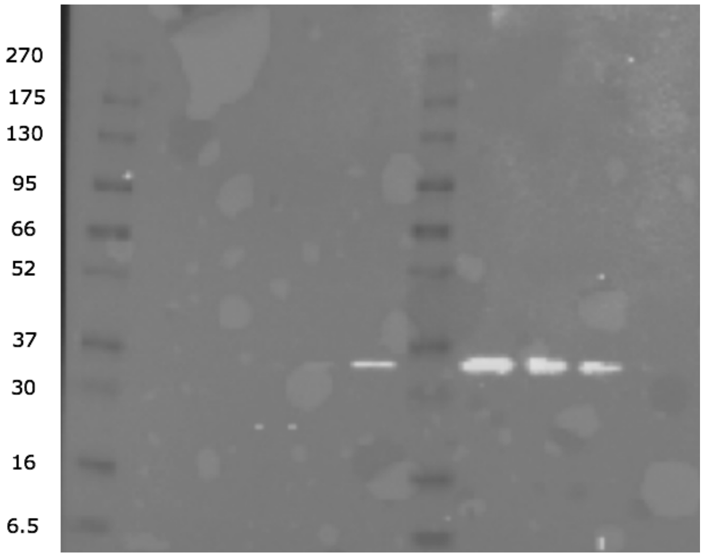

Supplementary Figure S3. **Full lenght image of Western blots depicted in Figure 3.** Full lenght image of Western blots depicted in Figure 3, developed with anti-cathepsin L primary antibody, on ovaries dissected from *Scaphoides titanus* females (ovaries from one insect per single lane) sampled at 20 days post injection of dsRNAs targeting green fluorescent protein (dsGFP) or ATP synthase β (dsATP), with construct St_dsATP1. The masses of protein standards migrated together with samples are indicated in kDa.
